# Treatment-related leukoencephalopathy in adults with central nervous system lymphoma: a retrospective analysis of 126 patients

**DOI:** 10.1007/s00277-024-05989-1

**Published:** 2024-09-13

**Authors:** Yasutaka Masuda, Katsuhiko Nara, Alice Fujii-Mori, Arika Shimura, Kazuki Taoka, Takeyuki Watadani, Ken Morita, Takehito Yamamoto, Mineo Kurokawa, Tappei Takada

**Affiliations:** 1 Department of Hematology and Oncology, Graduate School of Medicine, The University of Tokyo, Tokyo, Japan; 2 Department of Pharmacy, The University of Tokyo Hospital, Tokyo, Japan; 3 Clinical Application for Development of Therapy for Rare Disease, The University of Tokyo Hospital, Tokyo, Japan; 4 Department of Radiology, The University of Tokyo Hospital, Tokyo, Japan; 5 Department of Cell Therapy and Transplantation Medicine, The University of Tokyo Hospital, Tokyo, Japan

**Keywords:** Central nervous system lymphoma, Vitreoretinal lymphoma, High-dose methotrexate, Intrathecal methotrexate, Leukoencephalopathy

## Abstract

Neurotoxicity associated with high-dose chemotherapy and whole brain radiotherapy (WBRT) is one of major complications for patients with central nervous system lymphoma (CNSL). Here we determined the incidence and risk factors of treatment-related leukoencephalopathy (tLE) in a clinical setting. We retrospectively reviewed clinical and radiological findings of 126 patients with  (CNSL) treated with high-dose methotrexate with or without intrathecal methotrexate administration (IT MTX) and response-adapted WBRT. During the whole observation period with a median of 38.7 months, tLE was found in 33 patients, most of them asymptomatic, with the median time to development 3.0 months, and the cumulative incidence reaching 29.2% (95% confidence interval, 20.6–38.2%) two years post chemotherapy. By multivariable analysis, IT MTX was identified as the only one significant risk factor (hazard ratio, 4.50; *P* < 0.001), and the number of IT MTX was associated with the increased incidence and severity of tLE. These findings highlight the frequent neurological complications associated with CNS-directed therapy and confirm the neurotoxicity of IT MTX.

**Supplementary Information:**

The online version contains supplementary material available at 10.1007/s00277-024-05989-1.

## Introduction

Recognizing central nervous system (CNS) complications associated with cancer treatment is vital, as the range of cancer therapies with unique CNS toxicities is expanding [1]. In the last few decades of clinical trials exploring the optimal therapeutic approaches for primary CNS lymphoma (PCNSL), neurotoxicity as a consequence of therapy has been identified as an inevitable complication in a subset of patients [2–4]. Gavrilovic et al. have reported the incidence of neurotoxicity in patients treated with high-dose methotrexate (HD-MTX)-based combination chemotherapy and intra-Ommaya MTX with/without 45 Gy whole brain radiotherapy (WBRT) as a part of initial or salvage therapy [3]. In this report, 26% of patients who received 45 Gy WBRT developed clinically identified neurotoxicity defined as progressive neurologic or cognitive impairment as documented on serial clinical examinations, while only one out of 22 without 45 Gy WBRT developed neurotoxicity. Similarly, HD MTX combined with ifosfamide resulted in 26% incidence of clinically identified neurotoxicity as documented in serial clinical examinations, which increased to 49% when 45 Gy WBRT was added to chemotherapy [4]. In an attempt to mitigate the risk of treatment-related neurotoxicity, response-adapted reduction of the radiation dose was investigated [5]. In that protocol, the dose of WBRT was decreased to 23.4 Gy in patients who achieved CR after induction. When assessed with neuropsychological batteries, minimal neurotoxicity and even improved cognitive skills, specifically executive functions as evaluated by Trail Making Test and Brief Test of Attention, were observed following reduced-dose (< 30 Gy) WBRT [5–7].

Although evidence is mounting on the neurological complications of CNSL treatment in different therapeutic regimens, the incidence and risk factors of neurotoxicity in a clinical setting are largely undefined in part due to the rarity of the disease. Addressing this knowledge gap is particularly important for patients treated with regimens that include response-adapted WBRT, aimed at reducing treatment-related neurotoxicity. We have adopted HD MTX-based chemotherapy combined with response-adapted WBRT [5] for the treatment of primary and secondary CNS lymphoma. Further, we have validated its efficacy for vitreoretinal lymphoma [8, 9]. To shed light on this issue, we analyzed a large cohort of patients with CNS lymphoma treated with HD MTX with or without intrathecal MTX administration (IT MTX) and response-adapted WBRT at our institution. Importantly, prior studies adopted different definitions of neurotoxicity, making it difficult to appreciate the true scope of neurotoxicity. Here we specifically focused on treatment-related leukoencephalopathy (tLE), which could be chronic and might have lasting neurological consequences [10].

## Methods

### Patients

This is a single center, retrospective study involving consecutive patients retrospectively recruited who underwent rituximab, MTX, procarbazine, and vincristine (R-MPV) or MPV (collectively called “R-MPV” hereafter) therapy at the Department of Hematology and Oncology, The University of Tokyo Hospital between January 1st, 2008, and October 1st, 2020. The following patients were excluded: (1) patients who received only a single cycle of R-MPV, (2) patients who was initiated R-MPV in other hospitals, and (3) patients who were not evaluated with brain magnetic resonance imaging (MRI) both before and after R-MPV.

### Chemotherapy

R-MPV was conducted as per protocol presented in the original article [5] and with institutional modifications. In patients with positive cerebrospinal fluid (CSF) cytology, MTX 12 mg was administered via Ommaya reservoir in the original protocol [5], however at our institution, MTX 15 mg + prednisolone 20 mg was intrathecally administered once beyond day 5 of each cycle until CSF cytology become negative. For patients with vitreoretinal lymphoma, intravitreal injection of MTX and the systemic R-MPV chemotherapy were performed in parallel as previously described [9]. Treatment algorisms beyond R-MPV could be modified at the discretion of attending physicians. Before May 2020, treatment sequence was generally constructed as follows: after five cycles of R-MPV, patients were evaluated for response; if complete response was observed on brain MRI, patients proceeded to receive reduced-dose (23.4 Gy/13 Fr) WBRT and then to two cycles of high-dose cytarabine (HD AraC; 3000 mg/m^2^ × 2 days). If stable or progressive disease was observed, patients received full-dose (45 Gy/25 Fr) WBRT and then HD AraC. If partial response was observed, patients received two additional cycles of R-MPV and then reduced- or full-dose WBRT in cases with complete or partial/stable/progressive response after R-MPV, respectively. If apparent progression of the disease was observed or patients’ general status exacerbated during the initial five cycles of R-MPV, R-MPV was suspended or terminated at the discretion of attending physicians. From May 2020, upfront autologous stem cell transplantation conditioned with busulfan and thiotepa was considered for patients who achieved complete or partial response after induction R-MPV. This is because thiotepa has become available since 2019 in Japan. Dose of intravenously administered drugs were attenuated as per institutional guidelines. Longitudinal brain MRI to assess chemotherapy efficacy was conducted in all patients after completion of R-MPV.

### Data collection

For patients who met the inclusion criteria, the laboratory data and the following clinical data were obtained from the medical record: age, sex, body mass index, Eastern Cooperative Oncology Group Performance Status [11], pathological disease type, nervous system disease site, disease status, CSF cytology, whether IT MTX was performed before or during R-MPV, and laboratory data (complete blood count and biochemical tests). Clinical and laboratory data at the initiation of first cycle of R-MPV chemotherapy was obtained. Additionally, therapeutic drug monitoring of MTX during R-MPV cycles, preceding and following chemotherapy regimens, and dose of chemotherapy were also obtained from the record. For patients with tLE, attending physicians’ judgement regarding neurocognitive impairment based primarily on their impression. If necessary, cases were consulted to neurologists for further assessment.

### Definitions

Evaluation of leukoencephalopathy was performed on the brain MRI obtained primarily for the assessment of treatment efficacy at some point after the initiation of R-MPV therapy. All brain MRIs obtained during the follow-up period were reviewed. Detection of leukoencephalopathy was performed by a radiologist. Newly identified leukoencephalopathy after the initiation of R-MPV therapy without suspicion of disease relapse was considered treatment-related. In principle, bilateral/symmetrical high intensity on T2-weighted image and T2 fluid-attenuated inversion recovery (T2-FLAIR) image without gadolinium enhancement was considered therapy-related leukoencephalopathy. Hyperintensity lesions on T2-weighted or T2-FLAIR images already existed before treatment were considered age-related changes and were not deemed related to therapy. Leukoencephalopathy was graded according to Common Terminology Criteria for Adverse Events (CTCAE, version 5.0) by a single board-certified radiologist (T.W.), who was blinded to the result of the study. Abnormality in the laboratory data were also graded according to CTCAE version 5.0.

### Statistical analysis

Fisher’s exact test was used for categorical data. Follow-up started on the index date, defined as the date when the last cycle of R-MPV finished or when the last IT MTX accompanying R-MPV was administered, whichever was later. When tLE occurred during R-MPV therapy, the day of the occurrence of tLE was defined as day + 0.1 from the index date. In that case, the number of IT MTX times were counted up to the time of tLE development.

Probability of cumulative incidence of tLE was analyzed by cumulative incidence methods, and the groups were compared using the Gray test [12]. Competing risk for tLE was death before the occurrence of tLE. An adjustment to *P* values based on Holm’s method was applied to *post hoc* pairwise comparisons among more than two groups (e.g. the number of IT MTX during R-MPV). Fine-Gray competing risk regression model was employed to identify the factors influencing cumulative incidence of tLE.

The probabilities of overall survival (OS) and event-free survival (EFS) were estimated according to the Kaplan-Meier method, and the groups were compared using the log-rank test. OS was defined as the time from index date until death from any cause. Event was defined as progression or relapse, initiation of a new anticancer treatment, or death from any cause without progression. Patients were censored at the last follow-up date, at the first day of the CNS-directed salvage chemotherapy, or at 60 months from index date, whichever was first. Disease relapse was diagnosed with imaging and/or histological findings. To evaluate the influence of tLE development on EFS, proportional hazard modelling was used, treating the development of tLE as a time-dependent covariate.

Univariable and multivariable analyses were performed to identify risk factors for tLE. Multivariable logistic regression analysis was conducted for factors with *P* values < 0.1 that was obtained from univariable logistic regression analysis. The effect of WBRT on cumulative incidence of tLE was analyzed by semi-landmark method; the effect of WBRT on the occurrence of tLE was analyzed among patients who were not censored by the first day of WBRT (landmark point). A landmark comparison group comprised patients who survived until day 27, which was the median days of the initiation of WBRT.

All tests were two-sided, and *P* < 0.05 was considered statistically significant. Statistical analyses and figure presentations were performed with EZR version 1.63 [13].

## Results

### Patient characteristics

A total of 142 patients underwent R-MPV therapy during the study period. Sixteen patients were excluded for the following reasons: six received only a single cycle of R-MPV due to disease progression or death, five initiated R-MPV therapy at other hospitals, and five were not evaluated with brain MRI. Finally, 126 patients were included in the analysis. Table 1 summarizes the baseline characteristics of the studied population. The median patient age was 68 years (range, 26–87 years). R-MPV was performed with the median of 5 cycles (range, 2–7 cycles) per patient. IT MTX was performed in 31 (24.6%) patients, and the median number of IT MTX in patients who was performed IT MTX at least once was 5 (range, 1–7). WBRT was performed in 95 (75.4%) patients following R-MPV in total, with 13 and 82 patients receiving full- and reduced-dose WBRT, respectively. Only two patients proceeded to autologous stem cell transplantation in the study period.

**Table 1:** Characteristics of studied patients

Characteristic		Value
Age [year], median (range)		68 (26–87)
Age [year] - no. (%)		
	< 70 yo	74 (58.7)
	≥ 70 yo	52 (41.3)
Sex - no. (%)		
	Male	69 (54.8)
	Female	57 (45.2)
BMI [kg/m^2^], median (range)		21.8 (14.2–34.6)
BMI [kg/m2] - no. (%)		
	< 20	36 (28.6)
	≥ 20	90 (71.4)
ECOG PS - no. (%)		
	0	61 (48.4)
	≥ 1	39 (31.0)
	Not confirmed	26 (20.6)
Disease type - no. (%)		
	DLBCL	81 (64.3)
	Others^a^	7 (5.6)
	Not determined^b^	38 (30.2)
Disease site - no. (%)		
	CNS	75 (59.5)
	Vitreoretinal only	41 (32.5)
	CNS and vitreoretinal	10 (7.9)
Disease status - no. (%)		
	Primary	92 (73.0)
	Secondary	34 (27.0)
CSF cytology - no. (%)		
	Class 1/2	83 (65.9)
	Class 3/4/5	31 (24.6)
	Not determined^b^	12 (9.5)
R-MPV chemotherapy as - no. (%)		
	1st line	92 (73.0)
	≥ 2nd line	34 (27.0)
MTX dose - no. (%)		
	Full	111 (88.1)
	Reduced	15 (11.9)
MTX TDM high - no. (%)		
	0	68 (54.0)
	≥ 1	58 (46.0)
IT MTX before R-MPV - no. (%)		
	Not performed	117 (92.9)
	Performed	9 (7.1)
IT MTX with R-MPV - no. (%)		
	Not performed	95 (75.4)
	Performed	31 (24.6)
WBRT after R-MPV - no. (%)		
	Not performed	31 (24.6)
	Performed	95 (75.4)
	< 30 Gy performed	82 (65.1)
	45 Gy performed	13 (10.3)
Laboratory data		
	Alb [g/dL], median (range)	3.7 (2.6–4.7)
	AST [U/L], median (range)	20.5 (7.0-274.0)
	Peak AST ≥ grade 3^c^ - no. (%)	62 (49.2)
	ALT [U/L], median (range)	20.5 (7.0-274.0)
	Peak ALT ≥ grade 3^c^ - no. (%)	41 (32.5)
	T-bil [mg/dL], median (range)	0.7 (0.2–1.7)
	Peak T-bil ≥ grade 3^c^ - no. (%)	14 (11.1)
	Cre [mg/dL], median (range)	0.7 (0.3–1.1)
	Ccr [mL/min], median (range)	78.0 (42.7–198.9)

ALT, alanine aminotransferase; AST, aspartate aminotransferase; BMI, body mass index; CCr, creatine clearance; CNS, central nervous system; Cre, creatinine; CSF, cerebrospinal fluid; DLBCL, diffuse large B-cell lymphoma; ECOG PS, Eastern Cooperative Oncology Group Performance Status; Hb, hemoglobin; IT, intrathecal administration; MTX, methotrexate; R-MPV, rituximab, MTX, procarbazine, and vincristine therapy; T-bil, total bilirubin; TDM, therapeutic drug monitoring; and WBRT, whole brain radiation therapy

a, Other cases include follicular lymphoma, Burkitt lymphoma, lymphoplasmacytic lymphoma, anaplastic large cell lymphoma, and mycosis fungoides.

b, Unconfirmed cases include cases where diagnostic examinations were not performed or were poor sampling, and cases where the diagnosis or evaluation were ambiguous.

c, See text for definition.

### Incidence, timing, and characteristics of tLE

Median follow-up period was 38.7 months (range, 0.03–60 months), and MRI was captured with a median of 6 times (range, 1–21 times) per patient from the initiation of R-MPV to the end of follow-up. At 5 years, EFS rate was 54.6% (95% confidence interval [CI], 44.3–63.8%) and OS rate was 92.5% (95% CI, 84.8–96.4%).

**Fig. 1:**
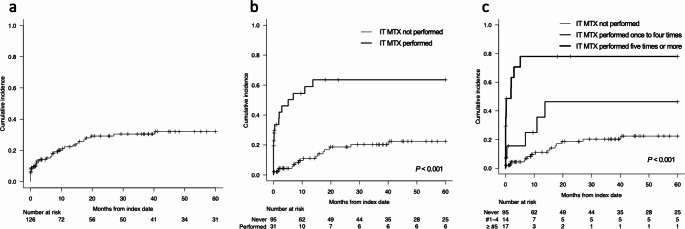
Cumulative incidence of tLE. Cumulative incidence of tLE in all studied patients (**a**) and when stratified according to the number of IT MTX times during R-MPV (**b**, **c**)

The cumulative incidence of tLE was 15.1% (95% CI, 9.3–22.2%) at 6 months, 29.2% (95% CI, 20.6–38.2%) at 2 years, and 32.0% (95% CI, 22.9–41.5%) at 5 years (Fig. 1a). MRI was captured with a median of 7 times (range, 2–20 times) and 6 times (range, 1–21 times) per patient with or without tLE from the initiation of R-MPV to the end of follow-up, respectively. During the whole observation period, tLE was found in 33 (26.2%) out of 126 patients, and median time from index date to tLE detection was 3.0 months (range, 0.003–40.8 months). tLE was detected during R-MPV in 7 patients and without WBRT exposure in 15 patients (including 9 patients who were not treated with WBRT after R-MPV). CTCAE grading of the 33 cases of tLE were grade 1 in 8 cases, grade 2 in 12, grade 3 in 11, and grade 4 in 2. No cases were considered disseminated necrotizing leukoencephalopathy. Review of the clinical record identified eight patients with clinically significant neurocognitive impairment subsequent to tLE development. They presented with forgetfulness and/or gait instability that aetiology other than tLE could reasonably be excluded. Half of them had tLE of grade 3 or more. Two cases were further consulted to neurologists, and in these case, Revised Hasegawa Dementia Scale test, which is routinely used for cognitive assessment in Japan (total score 30, 20 or lower is considered dementia) [14, 15] was 3, meaning severely impaired, in one patient and 25, meaning mildly impaired, in the other. Two of eight patients had been exposed to full-dose WBRT, and five of them to reduced-dose WBRT. IT MTX had been performed at least once in five out of the eight patients. At the last MRI during the follow-up in patients with tLE, radiographic manifestations remained in all of the 33 patients.

EFS of patients who developed tLE was not significantly different from those without tLE by univariable analysis (HR, 1.07; 95% CI 0.50–2.27; *P* = 0.87; Supplementary Table 1). OS rate was also comparable regardless of the development of tLE by multivariable analysis (HR, 2.72; 95% CI, 0.58–12.73; *P* = 0.2).

### Risk factors for tLE

To identify risk factors of tLE, various clinical variables were subjected to univariable and multivariable analysis (Table 2). Univariable analysis identified class 3/4/5 in CSF cytology (hazard ratio [HR], 3.11; 95% CI, 1.51–6.39; *P* = 0.002) and IT MTX during R-MPV (HR, 5.46; 95% CI, 2.72–10.94; *P* < 0.001) as only variables with *P* value below the threshold of significance as per the Bonferroni method of adjustment for multiple testing (*P* < 0.05/17 = 0.003). Other variables with at least borderline significance (*P* < 0.1) were disease in vitreoretinal lesions (HR, 0.38; 95% CI, 0.17–0.84; *P* = 0.017) and peak total bilirubin ≥ CTCAE grade 3 (HR, 3.02; 95% CI, 1.38–6.58; *P* = 0.006). When these variables, except for CSF cytology to avoid multicollinearity with IT MTX during R-MPV, were subjected to multivariable analysis, IT MTX during R-MPV was identified as a sole independent variable for tLE (HR, 4.50; 95% CI, 1.84–10.99; *P* < 0.001). Concomitantly, 5-year cumulative incidence of tLE was 22.4% (95% CI, 13.3–33.0%) and 63.5% (95%CI, 40.5–79.6%) in patients without and with IT MTX during R-MPV, respectively (Fig. 1b; *P* < 0.001).

**Table 2:** Risk factors for tLE

Variables		Univariable analysis		Multivariable analysis	
		HR (95% CI)	*P* values	HR (95% CI)	*P* values
Age [year]					
	< 70 yo	1			
	≥ 70 yo	0.76 (0.38–1.53)	0.44		
Sex					
	Male	1			
	Female	0.63 (0.31–1.26)	0.19		
BMI					
	< 20	1			
	≥ 20	1.25 (0.57–2.76)	0.57		
ECOG PS					
	0	1			
	≥ 1	1.49 (0.72–3.08)	0.28		
Disease site					
	CNS	1		1	
	Vitreoretinal only	0.38 (0.17–0.84)	**0.017**	0.84 (0.33–2.16)	0.72
	CNS and vitreoretinal	1.77 (0.75–4.18)	0.19	1.61 (0.61–4.20)	0.33
Disease status					
	Primary	1			
	Secondary	1.36 (0.64–2.88)	0.43		
CSF cytology					
	Class 1/2	1			
	Class 3/4/5	3.11 (1.51–6.39)	**0.002**		
R-MPV chemotherapy as					
	1st line	1			
	≥ 2nd line	1.67 (0.80–3.45)	0.17		
MTX dose					
	Full	1			
	Reduced	0.75 (0.23–2.46)	0.63		
MTX TDM high					
	0	1			
	≥ 1	0.87 (0.43–1.76)	0.71		
IT MTX before R-MPV					
	Not performed	1			
	Performed	0.89 (0.25–3.21)	0.86		
IT MTX with R-MPV					
	Not performed	1		1	
	Performed	5.46 (2.72–10.94)	**< 0.001**	4.50 (1.84–10.99)	**< 0.001**
Peak AST ≥ grade 3					
	No	1			
	Yes	0.70 (0.32–1.49)	0.35		
Peak ALT ≥ grade 3					
	No	1			
	Yes	1.18 (0.60–2.31)	0.64		
Peak T-bil ≥ grade 1					
	No	1		1	
	Yes	3.02 (1.38–6.58)	**0.006**	1.80 (0.67–4.81)	0.24
WBRT after R-MPV					
	Not performed	1			
	Performed	1.24 (0.28–5.49)	0.78		
	< 30 Gy performed	1			
	45 Gy performed	1.31 (0.33–5.24)	0.70		

ALT, alanine aminotransferase; AST, aspartate aminotransferase; BMI, body mass index; CSF, cerebrospinal fluid; ECOG PS, Eastern Cooperative Oncology Group Performance Status; IT, intrathecal administration; MTX, methotrexate; R-MPV, rituximab, MTX, procarbazine, and vincristine therapy; T-bil, total bilirubin; TDM, therapeutic drug monitoring; tLE, treatment-related leukoencephalopathy; and WBRT, whole brain radiation therapy

To probe further the association between IT MTX and tLE development, we evaluated the incremental effect of IT MTX times on the development of tLE. We classified the number of IT MTX times during R-MPV into three groups (group 1, not performed; group 2, performed once to four times; and group 3, five times or more) and assessed its stratified impact on the cumulative incidence and severity of tLE (Fig. 1c; Table 3). Development of tLE tended to show incremental increase according to the number of IT MTX times in terms of hazard ratio and 5-year cumulative incidence, although hazard ratio of group 2 vs. group 1 was nonsignificant. *P* values for 5-year cumulative incidence between group 1 and group 2, group 2 and group 3, and group 1 and 3 were 0.049, 0.039, and < 0.001, respectively. Severity of tLE graded according to CTCAE was also associated with the number of IT MTX times, with overall *P* = 0.003.

**Table 3:** Incidence and severity of tLE stratified by the number of IT MTX times during R-MPV

		Cumulative incidence				CTCAE grade					
	No.	HR (95% CI)	*P* values	5-year, %	*P* values	Total	1	2	3	4	*P* values
Group 1	95	1		0.22 (0.13–0.33)	**< 0.001**	16	8	6	2	0	**0.003**
Group 2	14	1.64 (0.65–4.12)	0.3	0.46 (0.14–0.74)		5	0	1	4	0	
Group 3	17	8.52 (3.65–19.87)	**< 0.001**	0.78 (0.43–0.93)		12	0	5	5	2	

CTCAE, Common Terminology Criteria for Adverse Events, version 5.0; IT MTX, intrathecal administration of methotrexate; R-MPV, rituximab, MTX, procarbazine, and vincristine therapy; and tLE, treatment-related leukoencephalopathy

## Discussion

In the present study, we characterized tLE associated with R-MPV treatment in 126 adult patients with CNS lymphoma with or without vitreoretinal involvement. We revealed that tLE after R-MPV was a frequent complication and was strongly associated with IT MTX during R-MPV.

We found that tLE occurred as early as during chemotherapy, prior to WBRT, and its incidence reaching approximately one-third of the patients two years after the index date (Fig. 1). In contrast to the delayed, clinically significant neurotoxicity observed years after chemotherapy in earlier trials involving full-dose WBRT [3, 4], several studies have reported that white matter changes post-PCNSL-targeted therapy begin to occur more acutely [6, 7, 16–19]. Subset of patients treated with combined modality chemotherapy plus reduced-dose WBRT showed an increase in white matter lesions immediately following chemotherapy [6, 7]. Furthermore, white matter changes developed in patients while receiving combined intravenous and intraventricular administration of methotrexate and cytarabine [18, 19]. These results were, however, with small sample sizes, making it difficult to ascertain the incidence and timing of treatment-related radiographic changes. Our findings with large number of patients revealed that tLE could develop relatively acutely in response to CNS-directed therapy, with the median time to tLE detection 3.0 months. Clinically, it is important to differentiate T2-FLAIR hyperintensity lesions from tumor lesions because the tumor sites could also be non-enhancing [20].

Risk factors for neurotoxicity is incompletely validated in literature. In patients with PCNSL treated with high-dose chemotherapy, combined use of WBRT was identified by multivariable analysis as the sole factor for clinically identified neurotoxicity, which presented as cognitive impairment that eventually fulfilled criteria for overt dementia [21]. In clinical trials, increased risk of clinically identified neurotoxicity were observed in patients who received full-dose WBRT, especially in older patients [2, 3]. These results have underscored the role of WBRT in inducing neurotoxicity, and thus regimens with reduced-dose WBRT have been explored [5, 6]. Here we extended these results to propose that IT MTX is a significant contributor to tLE development and exacerbation. We also showed a trend toward more frequent and severe tLE as the number of IT MTX increases. Conversely, out of 33 patients who developed tLE, 15 patients had no history of WBRT, and WBRT was not identified as significant risk factor for tLE. Our results are clinically relevant especially because the role of IT MTX is unclear with conflicting data from previous studies [22, 23]. Many guidelines now reserve IT MTX for patients with positive CSF cytology or leptomeningeal lesions, with low level of evidence [24–26]. The benefit of IT MTX should therefore cautiously be weighed against the potential role of IT MTX in causing LE.

There exist some reports on the transient LE and associated neurological deficits occurring subsequent to IT MTX [27]. However, chronic effect of IT MTX is still not defined, and it is therefore obscure whether IT MTX per se or IT MTX combined with intravenously administered HD MTX is responsible for tLE. Of note, MTX retains longer in the CSF when HD MTX is also administered, because significant amounts of systemically administered MTX enter the CSF [28]. Although decreased clearance of serum MTX was not significantly associated with tLE in our series, it is possible that systemically administered MTX might have augmented the neurotoxicity of IT MTX to some extent. Evaluation of MTX clearance in the CSF in patients receiving R-MPV with or without IT MTX could aid establish the causal role of MTX in inducing tLE.

Interestingly, many of the patients with tLE in our series were asymptomatic; eight out of 33 patients with tLE were identified with clinically significant neurocognitive impairment by chart review. Mild cognitive impairment might have been underestimated because we did not perform standardized cognitive screening. Classically the clinical correlates of early neurotoxicity of IT MTX as well as IV HD MTX are seizures, stupor, and altered consciousness [29], which were not observed in our patients. The asymptomatic nature of white matter changes post-CNS-directed therapy has been documented in several studies, specifically those omitting full-dose WBRT [7, 18, 30]. Supporting this, 30 Gy WBRT as consolidation to MTX-based chemotherapy has been shown to have negligible impact on neurocognitive functioning as evaluated by test batteries for attention/executive functioning, information processing, memory, and motor speed, and the extent of white matter abnormality had statistically significant but clinically small association with neurocognition [31]. The clinical significance of white matter changes after MTX exposure is still unclear considering the discrepancy between symptoms and MRI findings [32]. However, long-term implications are still not determined in patients with CNS lymphoma.

This study has some limitations. First, MRI was conducted primarily for the assessment of the disease and was not intentionally aimed for the detection of tLE. Second, the neurological symptoms of tLE were not evaluated with the standardized method and its presence was reported solely at the discretion of the attending physicians. These limitations might have led to the underestimation of the true incidence and the severity of tLE. In our study, the development of tLE was not associated with EFS nor OS. However, long-term assessment of neurocognitive skills and quality of life would be necessary to accurately appraise the tolerability of CNS complications. Third, our study was not aimed to evaluate the efficacy of IT MTX during R-MPV, therefore, whether the prognostic benefit of IT MTX outweighs the risk of tLE remains to be determined.

In conclusion, we showed that tLE after R-MPV was a frequent complication and was strongly associated with IT MTX during R-MPV. IT MTX-related neurotoxicity manifested as asymptomatic LE developing with relatively short latency in our series, whereas WBRT-related neurotoxicity manifests clinical deterioration with a delay at onset [21], although with response-adapted WBRT the neurocognitive skills would be preserved [7]. Our observations of IT MTX-related tLE echo with the assumption that chemotherapy-related neurotoxicity is a different entity from radiotherapy-related neurotoxicity [21]. Evaluation of the distinct and the synergetic effect of chemotherapy and radiotherapy on CNS and the long-term assessment of their neurological complications is still unknown and should merit further studies.

## Electronic supplementary material

Below is the link to the electronic supplementary material.

Supplementary Material 1

## Data Availability

Relevant data is available upon reasonable request to the corresponding author.
