# Supplementary material for: Treatment-related leukoencephalopathy in adults with central nervous system lymphoma: a retrospective analysis of 126 patients
Source: Ann Hematol. 2024 Sep 13;104(2):1095–104. doi: 10.1007/s00277-024-05989-1 (PMC11971226; doi:10.1007/s00277-024-05989-1)
Supplement: Supplementary file 1 — Supplementary Material 1 [file 277_2024_5989_MOESM1_ESM.docx]

| **Supplementary Table 1. Factors affecting EFS and OS.** | | | | | | | | | |
| --- | --- | --- | --- | --- | --- | --- | --- | --- | --- |
| Variables | | EFS | |  | OS | | | | |
|  |  | Univariable analysis | |  | Univariable analysis | |  | Multivariable analysis | |
|  |  | HR (95% CI) | *P* values |  | HR (95% CI) | *P* values |  | HR (95% CI) | *P* values |
| Age [year] - no. (%) | |  |  |  |  |  |  |  |  |
|  | < 70 yo | 1 |  |  | 1 |  |  |  |  |
|  | ≥ 70 yo | 0.97 (0.55-1.74) | 0.93 |  | 0.56 (0.11-2.87) | 0.48 |  |  |  |
| Sex - no. (%) | |  |  |  |  |  |  |  |  |
|  | Male | 1 |  |  | 1 |  |  |  |  |
|  | Female | 1.18 (0.67-2.07) | 0.57 |  | 0.92 (0.21-4.10) | 0.91 |  |  |  |
| ECOG PS - no. (%) | |  |  |  |  |  |  |  |  |
|  | 0 | 1 |  |  | 1 |  |  |  |  |
|  | ≥ 1 | 1.19 (0.63-2.26) | 0.59 |  | 1.34 (0.26-6.95) | 0.73 |  |  |  |
| Disease type - no. (%) | |  |  |  |  |  |  |  |  |
|  | DLBCL | 1 |  |  | 1 |  |  |  |  |
|  | Others | 1.84 (0.57-5.92) | 0.31 |  | 0.00 (0.00-Inf) | 1.00 |  |  |  |
| Disease site - no. (%) | |  |  |  |  |  |  |  |  |
|  | CNS | 1 |  |  | 1 |  |  |  |  |
|  | Vitreoretinal only | 0.76 (0.42-1.39) | 0.37 |  | 0.00 (0.00-Inf) | 1.00 |  |  |  |
|  | CNS and vitreoretinal | 0.79 (0.28-2.19) | 0.65 |  | 1.44 (0.17-11.96) | 0.74 |  |  |  |
| Disease status - no. (%) | |  |  |  |  |  |  |  |  |
|  | Primary | 1 |  |  | 1 |  |  | 1 |  |
|  | Secondary | 2.11 (1.15-3.87) | **0.02** |  | 10.51 (2.03-54.47) | **0.005** |  | 9.34 (1.78-49.04) | **0.008** |
| CSF cytology - no. (%) | |  |  |  |  |  |  |  |  |
|  | Class 1/2 | 1 |  |  | 1 |  |  |  |  |
|  | Class 3/4/5 | 1.23 (0.65-2.32) | 0.53 |  | 1.46 (0.28-7.54) | 0.65 |  |  |  |
| tLE development | |  |  |  |  |  |  |  |  |
|  | No | 1 |  |  | 1 |  |  | 1 |  |
|  | Yes | 1.07 (0.50-2.27) | 0.87 |  | 3.66 (0.80-16.75) | **0.09** |  | 2.72 (0.58-12.73) | 0.20 |
| CSF, cerebrospinal fluid; ECOG PS, Eastern Cooperative Oncology Group Performance Status; EFS, event-free survival; OS, overall survival; and tLE, treatment-related leukoencephalopathy. | | | | | | | | | |
